# Imaging characteristics and treatment strategies of traumatic diaphragmatic hernia with aortic dissection: a report of 3 cases

**DOI:** 10.1186/s13019-023-02117-4

**Published:** 2023-01-27

**Authors:** Hao-Hao Wu, Shao-Song Tu, Fang-Biao Zhang

**Affiliations:** 1 Department of Emergency, Lishui Hospital of Zhejiang University, Lishui Municipal Central Hospital, Lishui, 323000 China; 2 Department of Cardiothoracic Surgery, Lishui Municipal Central Hospital, The Fifth Affiliated Hospital of Wenzhou Medical University, No. 289 Kuocang Road, Lishui, 323000 Zhejiang Province China

**Keywords:** Trauma, Diaphragmatic hernia, Aortic dissection, Imaging, Surgery, Case report

## Abstract

**Background:**

Traumatic aortic dissection with traumatic diaphragmatic hernia is a rare traumatic disease. The purpose of this article is to investigate the imaging characteristics and treatment strategies for traumatic diaphragmatic hernia with aortic dissection.

**Case presentation:**

The imaging and clinical data of 3 patients with traumatic diaphragmatic hernia combined with aortic dissection were analyzed retrospectively. Of the three cases, two were males, and one was female; their mean age was 52.7 years (range, 47–62 years). Plain chest CT scans revealed diaphragmatic hernia in 2 patients, but no traumatic aortic dissection was found. Diaphragmatic hernia repair was performed in all patients. Aortic dilatation was found during intraoperative exploration, and aortic dissection was confirmed by postoperative enhanced CT. One patient underwent stent implantation and recovered smoothly (Case 1). The other patient refused stent implantation and died of thoracic hemorrhage (Case 2). The third patient underwent preoperative enhanced CT to identify traumatic diaphragmatic hernia with aortic dissection (Case 3). Aortic covered stent implantation was performed immediately, and diaphragmatic hernia repair was performed at a selected time. The patient’s postoperative recovery was good.

**Conclusion:**

A preoperative plain chest CT scan indicated diaphragmatic hernia in major blunt thoracic trauma patients with a history of trauma and blurred periaortic spaces accompanied by hematocele and other imaging manifestations. Chest-enhanced CT should be performed to improve the diagnostic accuracy of aortic dissection.

## Introduction

Traumatic aortic dissection refers to a hematoma formed by blood in the aorta that has entered the middle layer of the aorta through an intimal tear caused by the trauma. It is characterized by rapid onset, rapid progression and high mortality [1]. It is often missed due to combination with organ injury in other parts of the body. Traumatic diaphragmatic hernia refers to herniation of abdominal organs such as the stomach, liver and spleen into the chest due to trauma caused by rupture of the diaphragm muscle; it can cause respiratory and circulatory dysfunction, abdominal organ obstruction, and other complications, seriously threatening the life of the patient [2]. Traumatic aortic dissection with traumatic diaphragmatic hernia is a rare and multiple traumatic disease in clinical trauma. Early diagnosis is difficult, and the condition is easily missed and has a high mortality rate. Patients admitted to the emergency department usually receive only plain chest CT scan examinations, often resulting in missed diagnosis of aortic dissection. Three such patients were admitted to Lishui Municipal Central Hospital between January 2019 and January 2021. This paper analyzed the imaging features of those patients and the strategies used in their treatment.

## Case presentation

Between January 2019 and January 2021, 3 patients with traumatic diaphragmatic hernia complicated with traumatic aortic dissection were admitted to Lishui Municipal Central Hospital. The clinical study of those cases and the publication of this manuscript and the accompanying images were approved by the Ethics Committee for Clinical Research of Lishui Municipal Center Hospital. Clinical information from hospital records, including age, sex, pathogenic factors, symptoms, course, preoperative examination, former medical history, associated injuries and diagnosis, was retrospectively reviewed. Detailed information on the 3 patients is shown in Table 1.

**Table 1:** Patient clinical features

	Case 1	Case 2	Case 3
Sex	Male	Female	Male
Age (years)	47	62	49
Pathogenic factors	Traffic injury	Traffic injury	Fall injury
Symptoms	Pain	Coma	Pain
Course of the disease	72 h	3 h	24 h
Preoperative examination	Enhanced CT	Plain CT scan of the chest	Plain CT scan of the chest
Former medical history	Hypertension, atrial fibrillation	Hypertension, Atrial fibrillation	None
Associated injuries	Pulmonary contusion	Pulmonary contusion	Pulmonary contusion
	Pericardial rupture	Rib fracture	Rib fracture
	Lower limb fracture	Pelvic fractures	Lower limb fracture
	Spleen injury	Scapula fracture	Lumbar fractures
Diagnosis	Yes	No	No

*Case 1*

**Fig. 1:**
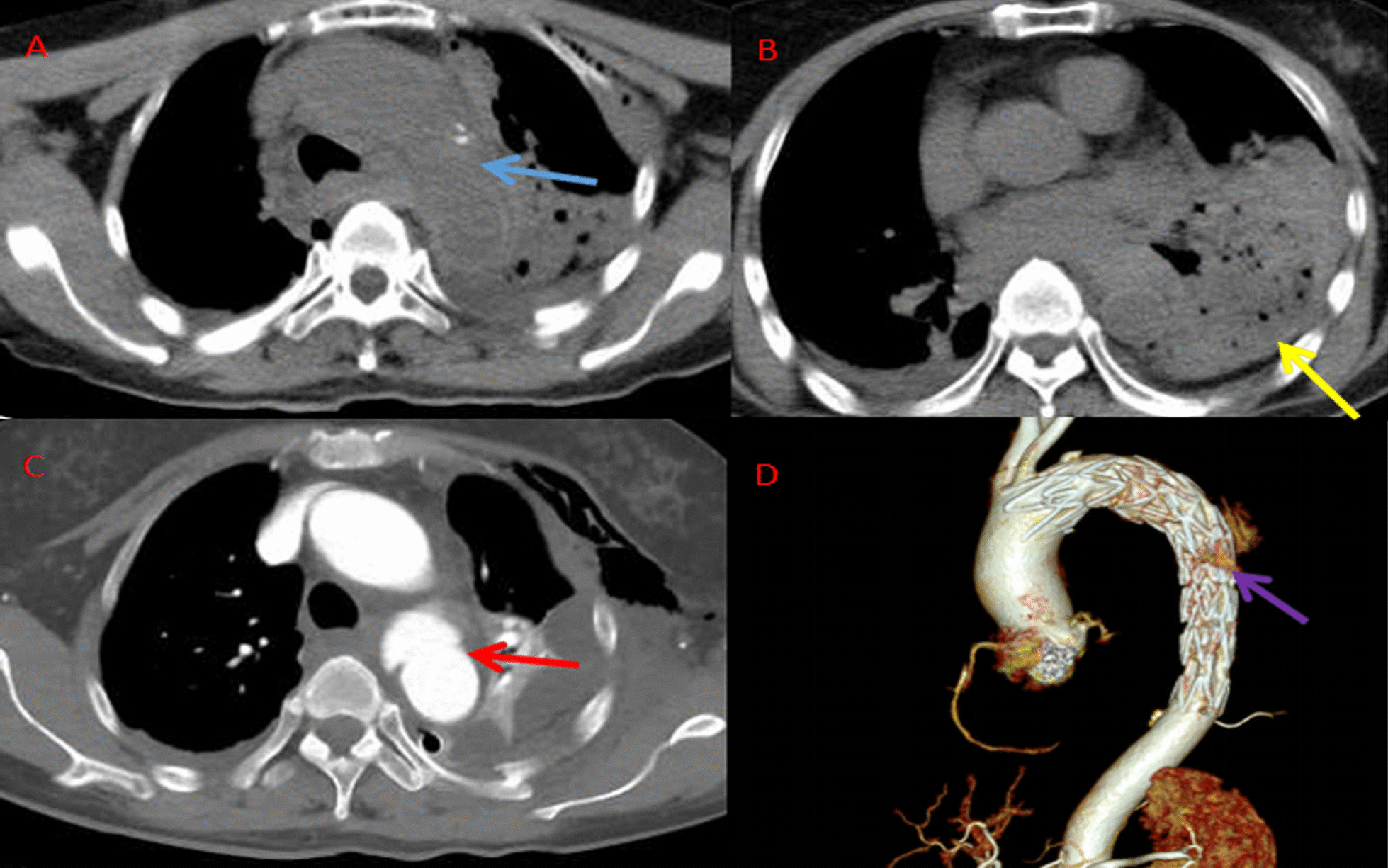
Imaging scan of Case 1. **A**, **B** A plain CT scan of the chest indicated a blurred mediastinal space with effusion (blue arrow), gastric hernia in the thoracic cavity (yellow arrow), and mediastinal gas and effusion/hemorrhage, and left diaphragmatic hernia was considered. **C**, **D** Vascular CTA indicated a possibility of dissection of the descending aorta (red arrow, **C**), and femoral artery stent implantation with a thin mesh was performed (purple arrow, **D**)

The patient was admitted to our hospital with coma caused by a car accident for 3 h. After the injury, he fell into a coma and was given endotracheal intubation and ventilator-assisted breathing in the emergency room. The body temperature was 36.5 °C, breath was 18/min, heart rate was 80/min and blood pressure was 149/75 mmHg. Laboratory tests demonstrated serum levels of aspartate aminotransferase (AST) was 74 U/L (normal 13–35 U/L), alanine aminotransferase (ALT) was 36 U/L (normal 7–40 U/L). Hemoglobin was 103 g/L (normal range, 120–160 g/L), white blood cell count was 12700 cells/mL (normal range, 3500–9500 cells/mL) and platelet count was 740 cells/mL (normal range, 1000–3000 cells/mL). Creatinine was 50 mmol/L (normal range, 46–92 mmol/L) and urea nitrogen was 4.5 mmol/L (normal range, 2.5–6.1 mmol/L). International normalized ratio (INR) was 1.06 and albumin was 30.6 g/L (normal 40–55 g/L). Since the patient's condition was unstable and the enhanced scan required a long time, only head and chest and abdominal plain CT were completed. Plain head CT showed no obvious bleeding, and plain chest CT revealed mediastinal air effusion and traumatic diaphragmatic rupture (Fig. 1A, B). According to other findings, the major diagnoses on admission were traumatic diaphragmatic rupture, rib fracture, lung contusion, pelvic fracture, and scapula fracture. The fractures occurred mainly in the 2nd to 12th ribs. We performed diaphragmatic hernia repair. During the operation, it was found that the diaphragm rupture was located anteriorly, about 8 cm in length, and the stomach and omentum herniated into the left thorax. Aortic probe swelled, possible aortic dissection was considered, and vascular enhancement CT examination indicated the possibility of dissection of the descending aorta (Figure 1C). The final diagnosis was aortic dissection, Stanford type B. The patient was given aortic angiography and thin net via femoral artery stenting and aortic lumen isolation (Fig. 1D). During the operation, it was found that the rupture was about 1cm away from the left subclavian artery, so the stent-anchored area was located behind the left common carotid artery, resulting in left subclavian artery obstruction without causing discomfort to the patient. The length of hospitalization was 21 days. The patient recovered well.

*Case 2*

**Fig. 2:**
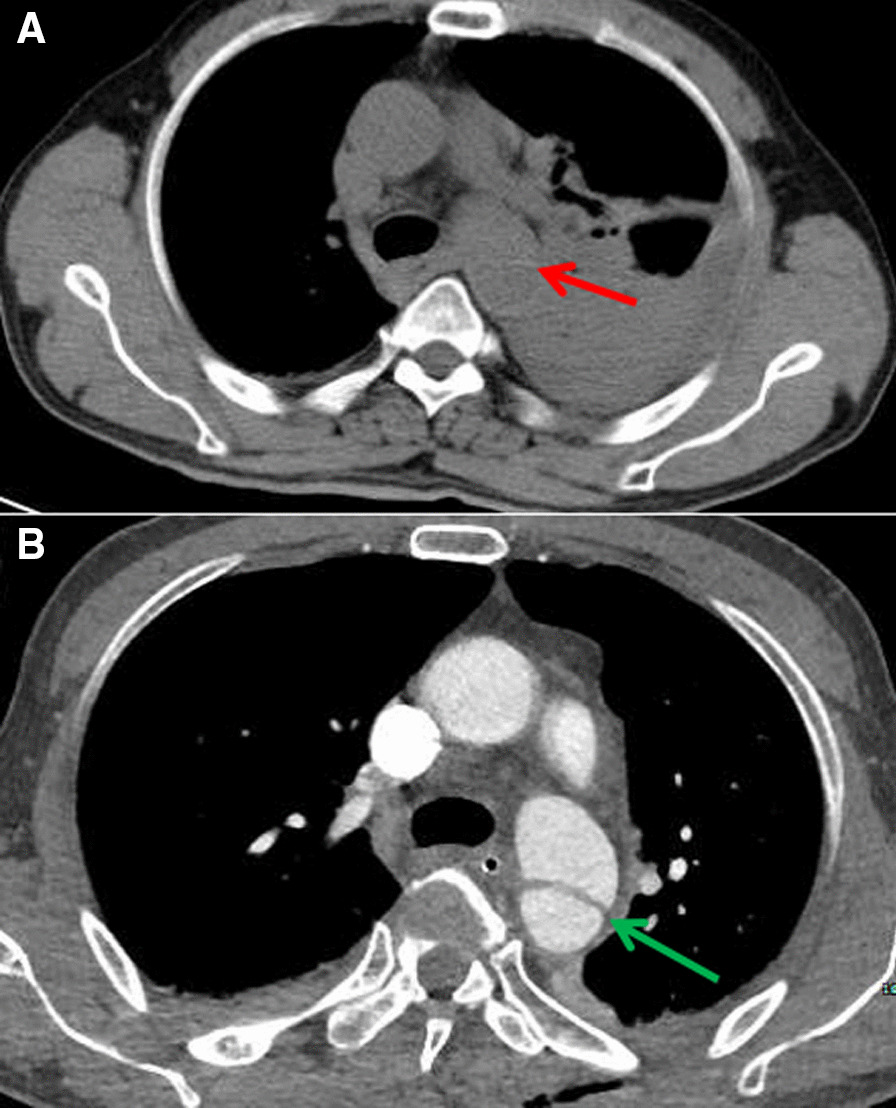
Chest CT imaging scan of Case 2. **A** The mediastinal space is slightly blurred, with a possible aortic boundary between the true and false lumen (red arrow). **B** Enhanced chest CT suggests aortic dissection (green arrow)

The second patient referred to our hospital due to experienced a fall from a height that had progressed over 12 h. After the injury, the patient reported many pains, mainly in the chest and abdomen, waist circumference, and right leg. After admission, the body temperature was 36.5 °C, breath was 20/min, heart rate was 100/min, and blood pressure was 137/85 mmHg. laboratory tests, including routine blood [hemoglobin, 103 g/L (normal range, 120–160 g/L); white blood cell count, 9600 cells/mL (normal range, 4000–10,000 cells/mL); platelet count, 1420 cells/mL (normal range, 1000–3000 cells/mL)], serum electrolyte [Na+, 139.0 mmol/L (normal range, 137–147 mmol/L); K+, 4.31 mmol/L (normal range, 3.5–5.3 mmol/L)]; glucose level [8.0 mmol/L (normal range, 3.9–6.1 mmol/L)]; liver function [glutamic- pyruvic transaminase, 65 U/L (normal range, 21–72 U/L)]; renal function [creatinine, 82 mmol/L (normal range, 55–105 mmol/L); urea nitrogen, 8.9 mmol/L (normal range, 3.2–7.1 mmol/L)], were most within normal limits. A plain chest CT scan showed a slightly blurred mediastinal space, left diaphragmatic discontinuity, gastric hernia into the left thoracic cavity, and no traumatic aortic dissection (Figure 2A). According to other findings, the main diagnoses on admission were traumatic diaphragmatic hernia, rib fracture, pulmonary contusion with infection, lumbar fracture, radial fracture, and tibiofibular fracture. The rib fractures occurred mainly in the 8th to 10th ribs. We performed diaphragmatic hernia repair. During the operation, the diaphragm rupture was found to be anteriorly located, approximately 10cm, and the stomach and omentum herniated into the left thoracic cavity. Aortic probe swelled, possible aortic dissection was considered, and enhanced chest CT indicated aortic dissection (Figure 2B). The final diagnosis was aortic dissection, Stanford type B. The patient refused aortic stent implantation and was transferred to a local hospital for treatment 2 days later. He died of sudden thoracic hemorrhage in the local hospital 7 days after discharge.

*Case 3*

**Fig. 3:**
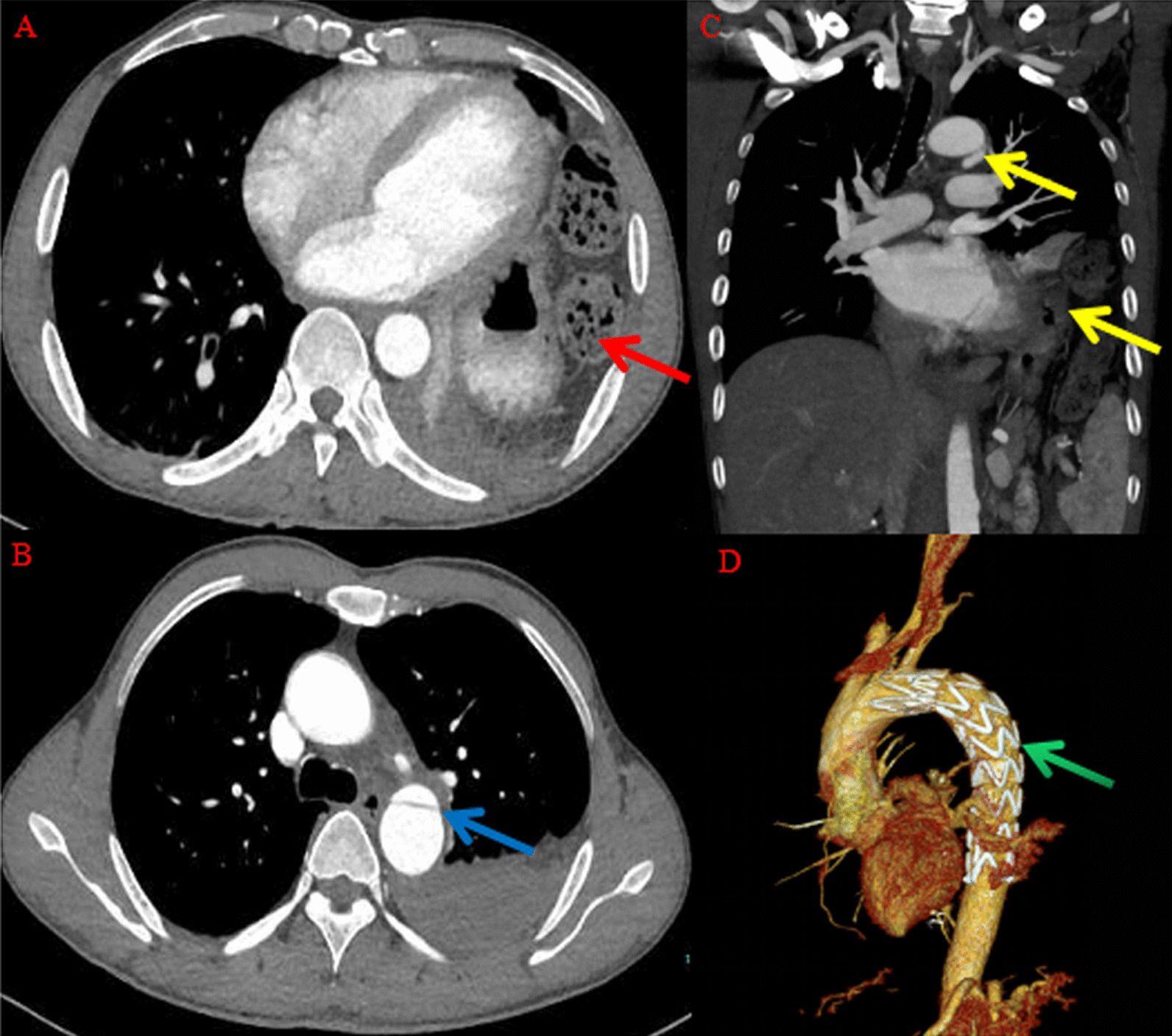
Vascular CTA imaging scan of Case 3. **A** Vascular CTA suggested herniation of the left abdominal contents into the thoracic cavity with consolidation of the left lower lobe (red arrow). **B** Intimomedial flap at the beginning of the descending aorta (blue arrow), and dissection was considered. **C** Vascular CTA showed traumatic diaphragmatic hernia with aortic dissection (Coronal, yellow arrow) **D** Femoral artery stent implantation with a thin mesh was performed (green arrow)

The third patient was involved in an automobile accident. The main manifestations after injury were pain in many locations, mainly in the chest and the left lower limb, accompanied by slight dizziness. He was treated at a local hospital. A plain chest CT scan during hospitalization indicated diaphragmatic rupture and blurred mediastinal space. Considering the mediastinal space blurring, chest enhanced CT was performed, and aortic dissection was found. On the third day of hospitalization, the patient was transferred to our hospital for treatment. The breath was 22/min, heart rate was 88/min and blood pressure was 154/104 mmHg. Laboratory tests demonstrated serum levels of aspartate aminotransferase (AST) 67 U/L (normal 13–35 U/L), alanine aminotransferase (ALT) 81 U/L (normal 7–40 U/L). Hemoglobin was 104 g/L (normal range, 120–160 g/L), white blood cell count was 9000 cells/mL (normal range, 3500–9500 cells/mL) and platelet count was 6300 cells/mL (normal range, 1000–3000 cells/mL). Creatinine was 52 mmol/L (normal range, 46–92 mmol/L) and urea nitrogen was 3.8 mmol/L (normal range, 2.5–6.1 mmol/L). According to the examination results, the main diagnoses were aortic dissection (Stanford type B), traumatic diaphragmatic hernia, spleen injury, lung contusion, and tibiofibular fracture. Preoperative vascular enhanced CT of the patient suggested that intimomedial flap at the beginning of the descending aorta. Dissection was considered, and the contents of the left abdominal cavity were herniated into the chest with consolidation of the left lower lobe (Fig. 3A–C). Oddly, this patient had no rib fractures. The patient with traumatic diaphragmatic hernia complicated with traumatic aortic dissection was immediately treated with aortography and transfemoral artery stent implantation with thin mesh (HT2424-080-1500, HT3430-160-2000) and aortic isolation (Figure 3D). Two days later, diaphragmatic hernia repair and pericardial repair and pleural hematocele removal were performed. During the operation, the diaphragm rupture was located in the front, about 7cm in length, and the stomach and omentum herniated into the left thorax. The length of hospitalization was 37 days. The patient recovered well.

## Discussion

Aortic injuries caused by high falls, traffic accidents and external compression injury are important causes of death [3]. Patients with traumatic aortic dissection often present with multiple injuries such as craniocerebral injury, multiple fractures, and abdominal organ injury, and the symptoms are easy to miss, leading to late diagnosis or misdiagnosis, with serious consequences. In assessment of complex injuries, early detection of a traumatic aortic dissection and early treatment are key to saving the patient's life.

The following aspects should be considered in the diagnosis and treatment of such trauma patients. (1) It is necessary to understand the basic disease. Hypertensive patients, in particular, should be very carefully assessed. Two patients in this group had hypertension prior to their injuries. (2) Attention should be given to clinical manifestations. Trauma patients often have multiple complications, and symptoms are easily masked. According to statistics, 76–96% of patients with aortic dissection have severe chest and back pain and discomfort. Therefore, more attention should be given to patients with combined back pain. (3) Attention should be given to the imaging findings obtained through plain chest CT scans. Although plain chest CT cannot diagnose aortic dissection, it can lead to imaging findings such as aortic dilatation, intermural hematoma, pleural effusion, and mediastinal gas effusion. Eighty-eight percent of patients with traumatic aortic dissection have pleural effusion. Once indicated, enhanced chest CT and other relevant examinations should be performed as soon as possible.

The treatment of patients with traumatic diaphragmatic hernia with traumatic aortic dissection is challenging. There are no analyses or summaries in the literature. In the authors' opinion, if the preoperative diagnosis is clear and the traumatic diaphragmatic hernia does not cause strangulated intestinal obstruction, bleeding or other complications, aortic surgery should be performed first to ensure the safety of diaphragmatic repair. Endovascular repair is a good method for the treatment of aortic dissection at present [4] and can be completed under local anesthesia to avoid cardiopulmonary bypass. In particular, the rupture of aortic dissection caused by trauma is often limited and provides a good anchoring area for coated stents. For patients with unclear preoperative diagnoses, aortic contusion and hematoma should be observed during diaphragm repair, and perioperative blood pressure should be strictly controlled to prevent intraoperative aortic rupture and even death. It is still debatable whether concurrent aortic vascular replacement plus diaphragmatic repair is feasible.

Traumatic diaphragmatic hernia combined with traumatic aortic dissection is not common among clinical trauma cases. Clinical symptoms may easily obscure each other and delay diagnosis and treatment. If not detected and treated in time, the condition often has serious consequences. Early diagnosis is the key factor in the treatment of severe traumatic disease. Two of the 3 patients described in this article had combined lower rib fractures. Lower rib fracture may provide additional evidence for the diagnosis of traumatic diaphragmatic hernia. Clinicians and radiologists should not ignore patients with lower rib fracture, past history of hypertension, blurred mediastinal space, mediastinal gas and effusion; in such cases, they should pursue timely application of enhanced chest CT and other examinations and should not rule out the possibility of aortic dissection.

## Conclusion

Automobile accidents, falls from heights, heavy extrusion and other traumas easily cause chest compound injuries. Traumatic aortic dissection is hidden and easily missed. This article reveals three important findings. First, for patients who have suffered major blunt thoracic trauma, especially when chest CT suggests mediastinal effusion, blurring around the aorta and other signs, enhanced chest CT examination should be performed as soon as possible. Second, if the patient does not have bleeding and necrosis caused by strangulated intestinal obstruction, it is suggested that aortic stent implantation be performed first, followed by diaphragmatic hernia repair. Third, if no aortic dissection is found before surgery and aortic dissection is possible during diaphragmatic hernia repair, chest enhanced CT should be performed as soon as possible and stent implantation should be considered once dissection is diagnosed.

## Data Availability

The data of this article are included within the article.
